# Predicting disease-associated microbes based on similarity fusion and deep learning

**DOI:** 10.1093/bib/bbae550

**Published:** 2024-10-30

**Authors:** Hailin Chen, Kuan Chen

**Affiliations:** School of Information and Software Engineering, East China Jiaotong University, Nanchang 330013, China; School of Information and Software Engineering, East China Jiaotong University, Nanchang 330013, China

**Keywords:** microbe-disease association, similarity fusion, graph convolution networks, jumping knowledge networks

## Abstract

Increasing studies have revealed the critical roles of human microbiome in a wide variety of disorders. Identification of disease-associated microbes might improve our knowledge and understanding of disease pathogenesis and treatment. Computational prediction of microbe-disease associations would provide helpful guidance for further biomedical screening, which has received lots of research interest in bioinformatics. In this study, a deep learning-based computational approach entitled SGJMDA is presented for predicting microbe-disease associations. Specifically, SGJMDA first fuses multiple similarities of microbes and diseases using a nonlinear strategy, and extracts feature information from homogeneous networks composed of the fused similarities via a graph convolution network. Second, a heterogeneous microbe-disease network is built to further capture the structural information of microbes and diseases by employing multi-neighborhood graph convolution network and jumping knowledge network. Finally, potential microbe-disease associations are inferred through computing the linear correlation coefficients of their embeddings. Results from cross-validation experiments show that SGJMDA outperforms 6 state-of-the-art computational methods. Furthermore, we carry out case studies on three important diseases using SGJMDA, in which 19, 20, and 11 predictions out of their top 20 results are successfully checked by the latest databases, respectively. The excellent performance of SGJMDA suggests that it could be a valuable and promising tool for inferring disease-associated microbes.

## Introduction

Microbes, which mainly refer to bacteria, but also include fungi and viruses, have been observed to live in and on human body sites, including urogenital tract, stomach, and skin [1]. The human body contains an estimated 350 trillion microbial cells [2]. Advances in metagenomics and metatranscriptomic analysis technologies have enabled the scientific community to explore the functions of human microbiome. Investigation into the human microbiome has revealed that they have a significant impact on our health. For instance, emerging evidence indicated that the gut microbiota is crucial in supporting health [3]. Another study found that a decrease in the amount of Faecalibacterium prausnitzii, an anti-inflammatory commensal bacterium, is linked to a higher chance of the recurrence of ileal Crohn’s disease (CD) [4].

Identification of human disease-associated microbes would provide a better understanding of disease etiology, which might lead to novel medical treatments [5]. Due to the significance of microbes in human health, researchers have searched published papers and established online databases [6, 7, 8] to systematically curate disease-associated microbes for further studies. Nevertheless, our knowledge of the microbe-disease associations has until now been limited. Meanwhile, it takes time and money to validate disease-associated microbes through *in vivo* studies. Computational predictions of such associations for further biomedical screening would be an excellent cost-effective alternative.

Till now, computational models to predict microbe-disease associations have garnered lots of research interest in bioinformatics field, and algorithms are constantly proposed with improved prediction accuracy [9]. These computational techniques can be mainly divided into three groups: network-based, matrix factorization-based, and machine learning-based.

Network-based methods apply graph theories to prioritize the unknown microbe-disease associations at the network level. For example, bi-random walk [10], KATZ measure combining network topology information [11] and network consistency projection in conjunction with label propagation [12] were utilized for inferring new microbe-disease associations. The network-based approaches can provide good interpretability of prediction results. However, there is still opportunity for improvements in their performance.

Matrix factorization-based approaches, usually under the low-rank assumption, have been widely used to recover user-item preference matrix in recommender systems [13]. Analogously, computational methods [14–16] were developed to apply matrix factorization to fill out the unknown elements in the original microbe-disease association matrix for new association predictions. Because of significant computational complexity of matrix operations, challenges would exist when these matrix factorization approaches are applied to large-scale datasets.

More recently, the fast advances in machine learning, especially deep learning, enable the development of efficient algorithms for the prediction of microbe-disease associations. For instance, adaptive boosting [17], back-propagation neural network [18] and deep sparse autoencoder neural network [19] were applied to infer new microbe-disease associations. These computational methods are receiving encouraging prediction results.

Despite of success of the above methods in identifying microbe-disease associations, some challenges should be tackled for better predictions. First, as biomedical technologies advance, more and more data features of microbes and diseases are available. Integrating these heterogeneous features for more reliable and accurate prediction is a challenging task. Second, biomedically confirmed negative samples cannot be obtained when using supervised learning methods for association prediction. Some proposed methods usually select negative samples randomly from the unlabeled microbe-disease pairs, in which noise exists and inaccurate results would be received. Finally, the successful applications of deep learning are encouraging us to develop more robust and precise algorithms to predict microbe-disease associations.

In this study, we develop an algorithm named SGJMDA based on similarity fusion using graph convolution networks and jumping knowledge networks for microbe-disease association predictions. We first collect four categories of similarities for microbes and diseases, respectively. A non-linear method is applied to fuse these similarities. Graph convolution networks and jumping knowledge networks are then used to extract features of microbes and diseases, respectively. Linear correlation coefficients between microbes and diseases are finally calculated as the prediction results. We comprehensively test and compare the performance of SGJMDA based on benchmark datasets and cross validations. We also conduct case studies to showcase the prediction ability of SGJMDA in real situations. With excellent performance received, we expect our method SGJMDA would be helpful for biomedical researchers in predicting microbe-disease associations.

## Materials and methods

### Datasets

In this study, we download the benchmark datasets from reference [19] for performance analysis. We give a brief explanation of the datasets below.

### Human microbe–disease associations

In reference [19], authors retrieved human microbe-disease associations from three existing databases (i.e. HMDAD [6], Disbiome [7] and Peryton [8]). After deleting the redundant data and information merging, 4499 experimentally validated microbe-disease associations, which contain 1177 microbes and 134 diseases, were collected as gold standard data. We use ${N}_m$ and ${N}_d$ to denote the numbers of microbes and diseases, respectively. Meanwhile, A$\in{R}^{N_m\times{N}_d}$ is used to represent the adjacency matrix of the microbe-disease associations, where ${N}_m\ $(=1177) is the number of rows (microbes) and ${N}_d$(=134) denotes the number of columns (diseases). A(i,j) = 1 indicates an association between microbe ${m}_i$ and disease ${\mathrm{d}}_j$ exists. Otherwise A(i,j) = 0.

### Similarity calculation and fusion

In reference [19], authors utilized four methods to compute similarities for microbes and diseases. Firstly, they calculated the semantic similarity(DS)of diseases. Based on this, the functional similarity(FS)of microbes was then derived. Subsequently, they further computed the cosine similarity (COS_MS，COS_DS), Gaussian interaction profile similarity(GIP_MS, GIP_DS), and sigmoid kernel function similarity(SIG_MS，SIG_DS)for both microbes and diseases. We download the similarities from reference [19].

As data from different sources can provide complementary information, while containing potential noise [20, 21], we apply a non-linear strategy, motivated by reference [20], to fuse these similarities for both microbes and diseases. Firstly, we standardize COS_MS, GIP_MS, and SIG_MS of microbes. Taking COS_MS as an example, the normalization process is computed as follows:


(1) \begin{equation*} COS\_ MS\left(i,j\right)=\left\{\begin{array}{@{}cc}\frac{COS\_ MS\left(i,j\right)}{2\ast{\sum}_{k\ne i} COS\_ MS\left(i,k\right)},& i\ne j\\{}\frac{1}{2},& i=j\end{array}\right. \end{equation*}

Set all diagonal elements of the matrix to 1/2, and the total sum of elements in each row is equal to 1. We can obtain $S{M}_{COS}$ by normalizing COS_MS. Meanwhile, we process GIP_MS and SIG_MS in the same way to obtain $S{M}_{GIP}$ and $S{M}_{SIG}$, respectively. Next, we use the KNN algorithm to calculate the local affinity $S{KNN}_{COS}$ for microbe $\mathrm{i}$ and microbe $j$ as follows:


(2) \begin{equation*} SKN{N}_{COS}\left(i,j\right)=\left\{\begin{array}{@{}cc}\frac{COS\_ MS\left(i,j\right)}{\sum_{k\in{N}_i} COS\_ MS\left(i,k\right)},& j\in{N}_i\\{}0,& otherwise\end{array}\right. \end{equation*}

${N}_i$ is the set of KNNs for a given node, where ${N}_i$ is equal to the total number of microbes divided by 10. Based on the principle that the closer the distance, the higher the similarity, we set the similarity between nodes far away from a given node to 0. Similarly, we can obtain $S{KNN}_{GIP}$ and $S{KNN}_{SIG}$.

We simultaneously update the three similarity networks as follows:


(3) \begin{equation*} S{M}_{COS}^{(t)}= SKN{N}_{COS}\times \left(\frac{\sum_{k\ne COS\_ MS}S{M}_k^{\left(t-1\right)}}{\mathrm{m}-1}\right)\times{\left( SKN{N}_{COS}\right)}^T, \end{equation*}

(4) \begin{equation*} S{M}_{GIP}^{(t)}= SKN{N}_{GIP}\times \left(\frac{\sum_{k\ne GIP\_ MS}S{M}_k^{\left(t-1\right)}}{\mathrm{m}-1}\right)\times{\left( SKN{N}_{GIP}\right)}^T, \end{equation*}

(5) \begin{equation*} S{M}_{SIG}^{(t)}= SKN{N}_{SIG}\times \left(\frac{\sum_{k\ne SIG\_ MS}S{M}_k^{\left(t-1\right)}}{\mathrm{m}-1}\right)\times{\left( SKN{N}_{SIG}\right)}^T, \end{equation*}

Among them, *m* represents the number of different similarity networks of microbes. Since we use three similarity networks, therefore *m* = 3. *K* is the selected microbe similarity network. *T* denotes the times of iterations. The similarity matrix *SM* is calculated as:


(6) \begin{equation*} SM=\frac{S{M}_{COS}^{\left(\mathrm{t}\right)}+S{M}_{GIP}^{\left(\mathrm{t}\right)}+S{M}_{SIG}^{\left(\mathrm{t}\right)}}{3}, \end{equation*}

When the condition $\frac{\left\Vert S{M}_k^{(t)}-S{M}_k^{\left(t-1\right)}\right\Vert }{\left\Vert S{M}_{\mathrm{k}}^{\left(\mathrm{t}-1\right)}\right\Vert }<{10}^{-6}$ is met, the iteration will end. We obtain $S{M}^{\prime }$ through the following equation:


(7) \begin{equation*} S{M}^{\prime }=\frac{SM+S{M}^T}{2}, \end{equation*}

Finally, we set a hyperparameter $\alpha$ to merge FS with $S{M}^{\prime }$ as follows:


(8) \begin{equation*} S{M}^{{\prime\prime} }=\alpha \ast S{M}^{\prime }+\left(1-\alpha \right)\ast FS, \end{equation*}

**Figure 1:**
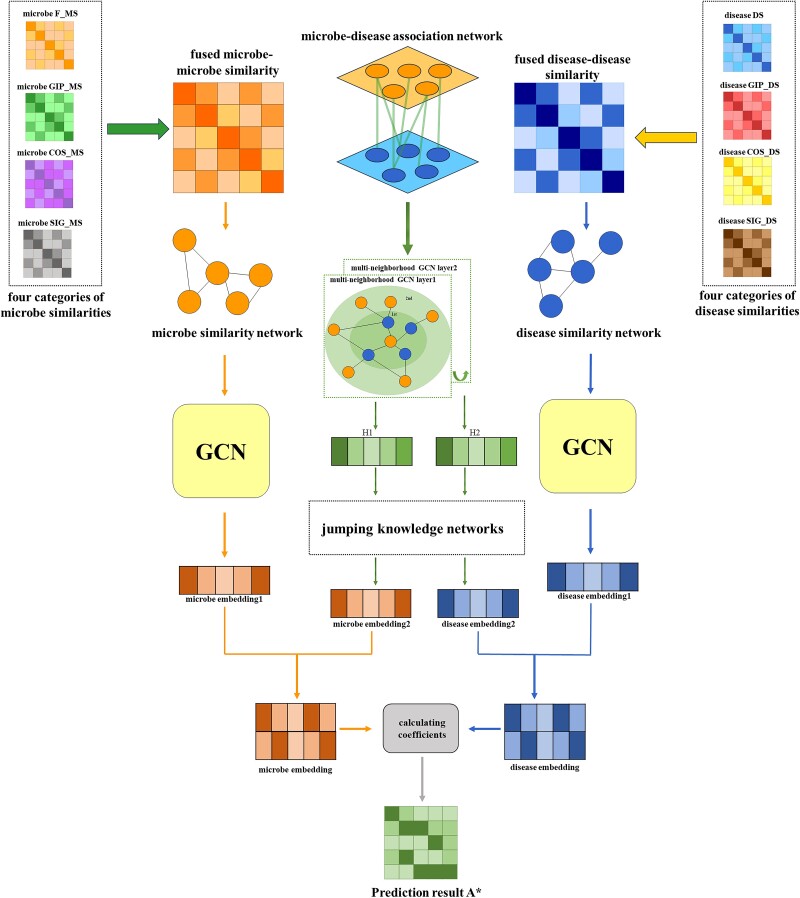
The workflow of SGJMDA in microbe-disease association inference.

The hyperparameter α is a weighting factor. $S{M}^{{\prime\prime} }$ represents the final result of fused similarity feature matrices. Similarly, we can use the above methods to obtain the similarity feature matrix $S{D}^{{\prime\prime} }$ of diseases.

### Method architecture

The architecture of SGJMDA for association prediction is presented below. The computational framework mainly consists of two parts. The first part is feature extraction and the other one is association prediction. We illustrate the workflow of SGJMDA in Fig. 1.

After similarity fusion, we apply GCN [22] for feature extraction for both microbes and diseases. The progressive spread rule formula of GCN used in our study is similar to reference [23], which is defined as follows:


(9) \begin{equation*} {H}^{\left(l+1\right)}=f\left({H}^{(l)},G\right)=\sigma \left({D}^{-\frac{1}{2}}G{D}^{\frac{1}{2}}{H}^{(l)}{W}^{(l)}\right), \end{equation*}

Where ${H}^{(l)}$ denotes the embeddings of nodes at the *l*-th layer, $D=\mathit{\operatorname{diag}}\left({\sum}_j{G}_{ij}\right)$ is the degree matrix of $G$, ${W}^{(l)}$ is the trainable weight matrix to the *l*-th layer and $\sigma \left(\cdot \right)$ is a non-linear activation function.

Taking microbes as an example, we define the input graph G as:


(10) \begin{equation*} G=\left[S{M}^{{\prime\prime}}\right], \end{equation*}

Then, our first layer GCN can be formulated as:


(11) \begin{equation*} {H}^{(1)}=\sigma \left({D}^{-\frac{1}{2}}G{D}^{\frac{1}{2}}{\left(S{M}^{{\prime\prime}}\right)}^{(0)}{W}^{(0)}\right), \end{equation*}

where ${W}^{(0)}\in{\mathbb{R}}^{N_m\times k}$ represents the first layer weight matrix, and *k* is used to change the dimension of the embedding. Due to the fact that our model only uses one layer of GCN in the homogeneous network, the feature output of microbes obtained through GCN is $S{M}^{{\prime\prime\prime}}\in{\mathbb{R}}^{N_m\times k}$. Similarly, we can obtain the feature output of diseases $S{D}^{{\prime\prime\prime}}\in{R}^{N_d\times k}$.

Meanwhile, inspired by MINIMDA [24], we construct microbe-disease heterogeneous networks to integrate mixed high-order neighborhood information to further obtain representation of microbes and diseases. We use the microbe-disease adjacency matrix and fully connected $S{M}^{{\prime\prime\prime} }$ and $S{D}^{{\prime\prime\prime} }$ for feature extraction as follow:


(12) \begin{equation*} {H}^{\left(l+1\right)}=\left\Vert{}_{i\in{K}^{\sigma }}\left({\left({\tilde{D}}^{-\frac{1}{2}}\tilde{G}{\tilde{D}}^{\frac{1}{2}}\right)}^i{H}^{(l)}{W}_i^{(l)}\right)\right., \end{equation*}

(13) \begin{equation*} \tilde{G}=\left[A\right], \end{equation*}

(14) \begin{equation*} {H}^{(0)}=\left[\begin{array}{c}S{M}^{{\prime\prime\prime}}\\{}S{D}^{{\prime\prime\prime}}\end{array}\right], \end{equation*}

where $K$ represents the power of ${\tilde{D}}^{-\frac{1}{2}}\tilde{G}{\tilde{D}}^{\frac{1}{2}}$, and $K=\left\{0,1,2,...,k\right\}$ denotes the order of neighbors for information propagation between features. When $k=2$, $K$ will be $\left\{0,1,2\right\}$, which means that the model will only receive information from the 0, 1st, and 2nd order neighborhoods.

In order to effectively aggregate the representation of intermediate layers to the final layer, we apply the mechanism of jumping knowledge (JK) network [25] to aggregates these different layers, which is calculated as follows:


(15) \begin{equation*} \tilde{H}={\sum}_{i=1}^l{\omega}_i{H}^{(i)}, \end{equation*}

where ${\omega}_i$ represents the weighting coefficient for feature aggregation in the $i$-th layer. The features $\tilde{SM}$ and $\tilde{SD}$ of microbes and diseases will then be obtained, respectively.

We use linear correlation coefficients to infer possible microbe-disease associations [26], which is computed according to the following equation:


(16) \begin{equation*} Corr\left({\lambda}_i,{\lambda}_j\right)=\frac{\left({\lambda}_i-{\mu}_i\right){\left({\lambda}_j-{\mu}_j\right)}^T}{\sqrt{\left({\lambda}_i-{\mu}_i\right){\left({\lambda}_i-{\mu}_i\right)}^T}\sqrt{\left({\lambda}_j-{\mu}_j\right){\left({\lambda}_j-{\mu}_j\right)}^T}}, \end{equation*}

where $${\lambda}_i\in \left[\begin{array}{@{}c@{}}S{M}^{{\prime\prime\prime}}\\{}\tilde{SM}\end{array}\right]$$ and $${\lambda}_j\in \left[\begin{array}{@{}c@{}}S{D}^{{\prime\prime\prime}}\\{}\tilde{SD}\end{array}\right]$$ are vectors representing the features of the $i$-th microbe and $j$-th diseases, respectively, and ${\mu}_i$and ${\mu}_j$ are the average values of ${\lambda}_i$ and ${\lambda}_j$.

Finally, we use the sigmoid function to reconstruct the microbe-disease matrix $\hat{A}$, according to the following formula:


(17) \begin{equation*} \hat{A}=\varphi \left( Corr\left(\left[\begin{array}{@{}c@{}}S{M}^{{\prime\prime\prime}}\\{}\tilde{SM}\end{array}\right],\left[\begin{array}{@{}c@{}}S{D}^{{\prime\prime\prime}}\\{}\tilde{SD}\end{array}\right]\right)\right), \end{equation*}

Optimization of model parameters is based on binary cross entropy loss (see equation 18):


(18) \begin{equation*} \mathcal{L}=-{\sum}_{\left(i,j\right)\in{\gamma}^{+}\cup \gamma -}\left[{A}_{ij}\ln \hat{A_{ij}}+\left(1-{A}_{ij}\right)\ln \left(1-\hat{A_{ij}}\right)\right], \end{equation*}

where ${\gamma}^{+}$ and ${\gamma}^{-}$ represent the sets of positive and negative instances used in training. $\left(i,j\right)$ indicates microbe $i$ and disease $j$. ${\hat{A}}_{ij}$ denotes the predicted score between microbe $i$ and disease $j$.

## Results

### Experimental setting

We use 5-fold and 10-fold cross-validations (5-CV and 10-CV) to evaluate the performance of our model. For 5-CV, we randomly divide the 4499 microbe-disease associations into five roughly equal parts, with four parts for training and the remaining one for testing. The similar steps are taken in the 10-CV tests. We further calculate AUC, AUPR, Recall, precision (Pre), accuracy (ACC) and F1-score as indicators for performance comparison.

### Hyperparameter analysis

Our method SGJMDA contains the following hyperparameters: (i) the proportion coefficient of feature fusion $\alpha$, (ii) the embedding dimension layer_size, (iii) the number of layers in multi-neighborhood GCN n, and (iv) the number of head nodes in multi-neighborhood GCN $k$. We analyse the impact of different parameter setting on prediction performance based on 10-CV using the benchmark datasets.

**Figure 2:**
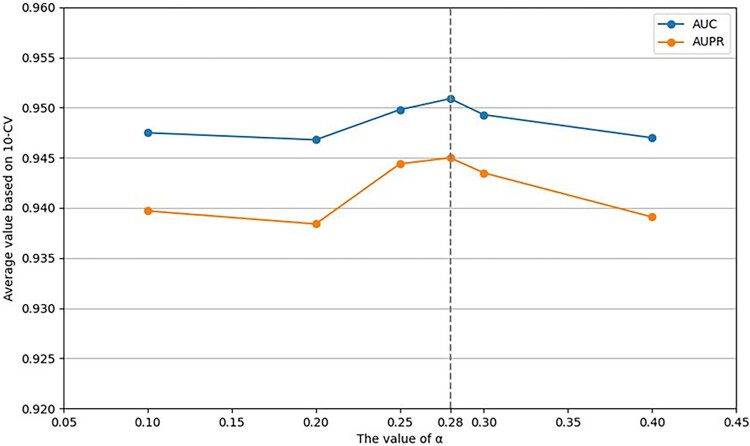
Performance analysis on the proportion coefficient $\alpha$.

Firstly, for the proportion coefficient of feature fusion $\alpha$, we select its value as 0.1, 0.2, 0.25, 0.28, 0.3 and 0.4 for comparison and find that when the proportion coefficient $\alpha$ of feature fusion equals 0.28, the model receives the best performance (see Fig. 2).

**Figure 3:**
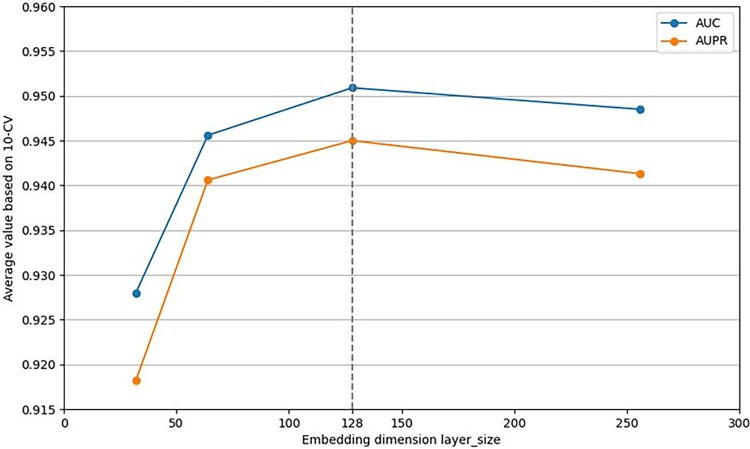
Performance analysis on the dimension of layer_size.

Secondly, we change the dimension of layer_size embedded in GCN and analyse its impact on prediction performance. As shown in Fig. 3, we set its value to be 32, 64, 128 and 256 and results show that when layer_size = 128, our model performs best.

**Figure 4:**
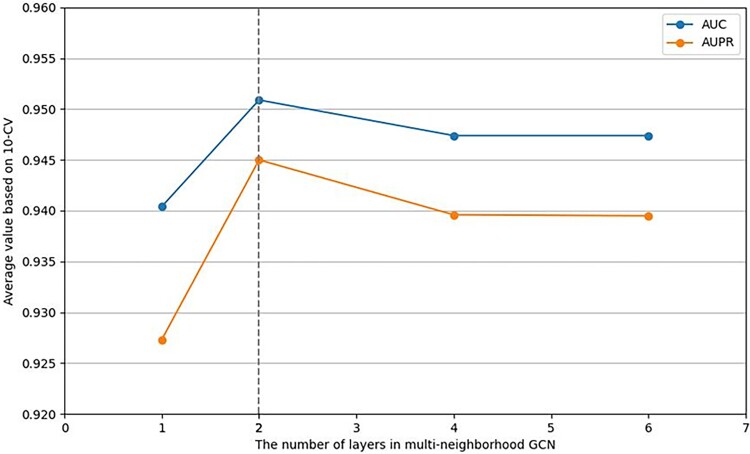
Performance analysis on the number of layers in multi-neighborhood.

Thirdly, we select the number of layers for multi-neighborhood GCN in our model to be 1, 2, 4, and 6. Figure 4 shows that when the number is 2, our model performances best.

**Figure 5:**
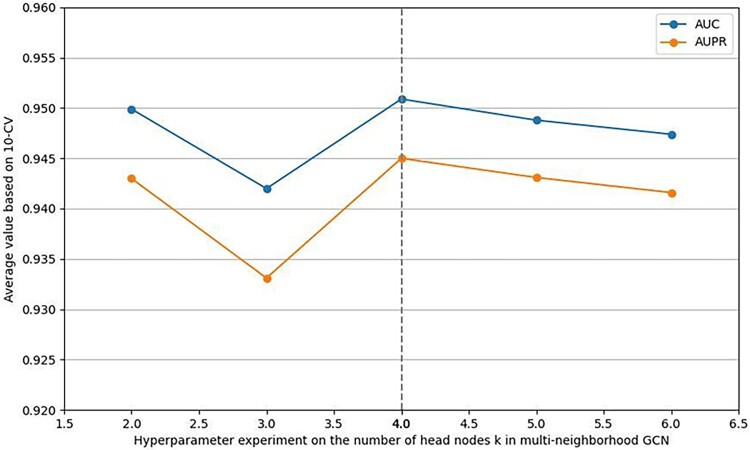
Performance analysis on the number of head nodes in multi-neighborhood.

Finally, we test the impact of the number of head nodes $k$ in multi-neighborhood GCN. We set its value as 2, 3, 4, 5, and 6 respectively. As shown in Fig. 5, when $k$ equals 4, our model receives its best performance.

Based on the above experimental tests, we set $\alpha$ to 0.28, layer_size to 128, n to 2 and $k$ to 4 in SGJMDA. In addition, we empirically set lr = 0.001, wd = 1e-5, and epoch = 2000 in our study.

### Ablation experiments

There are five key modules in our method SGJMDA: feature fusion, GCN fused with homogeneous networks, multi-neighborhood GCN, jumping knowledge and decoder. We remove each component from SGJMDA separately to investigate their impacts on prediction ability. Here are the five models we test and compare:

SGJMDA-SF model: We remove the feature fusion component used in this paper and replace it with averaging, while keeping the rest unchanged.

SGJMDA-Hom model: We preserve feature fusion, multi-neighborhood GCN, jumping knowledge and decoder, and replace GCN with fully connected networks.

SGJMDA-Het model: We remove multi-neighborhood GCN and jumping knowledge, while keep other modules unchanged.

SGJMDA-JK model: We remove the jumping knowledge module, leaving all other modules unchanged.

SGJMDA-Dec model: The decoder is replaced by utilizing fused matric of microbial feature and the transposition of fused matric of disease features for matrix multiplication. Subsequently, applying the sigmoid function to generate the final score matrix.

As shown in Table 1, the performance of SGJMDA-SF is inferior to SGJMDA, indicating that our non-linear fusion strategy can more effectively integrate features of microbes and diseases and achieve better prediction performance; The performance of SGJMDA-Hom is worse when compared with SGJMDA, indicating that homogeneous GCN can better learn the features of microbes and diseases; SGJMDA-Het is only slightly better than SGJMDA on Recall, while other indicators are lower, demonstrating that multi-neighborhood GCN also contributes to the embedding of microbes and diseases; The performance of SGJMDA-JK is worse than that of SGJMDA, suggesting that the using jumping knowledge can improve prediction performance; SGJMDA-Dec performs much worse than SGJMDA, indicating that the linear correlation coefficients are suitable to predict microbe-disease associations in our study.

**Table 1:** Results of ablation tests based on 10-CV

Method	AUC	AUPR	Accuracy	Precision	Recall	F1-score
SGJMDA SGJMDA-SF SGJMDA-Hom SGJMDA-Het SGJMDA-JK SGJMDA-Dec	**0.9509** 0.9295 0.9364 0.9495 0.9404 0.8433	**0.9450** 0.9297 0.9293 0.9410 0.9273 0.8650	**0.8914** 0.8608 0.8728 0.8908 0.8821 0.7912	**0.8677** 0.8548 0.8579 0.8628 0.8593 0.8107	0.9251 0.8695 0.8949 **0.9299** 0.9155 0.7633	**0.8951** 0.8620 0.8755 0.8945 0.8861 0.7853

### Comparison with other methods

We compare SGJMDA with six baseline methods (i.e. DSAE_RF [19], AMHMDA [27], MHCLMDA [28], MNNMDA [29], LRLSHMDA [30], and NTSHMDA [31]) using the same benchmark datasets and cross-validations.

**Figure 6:**
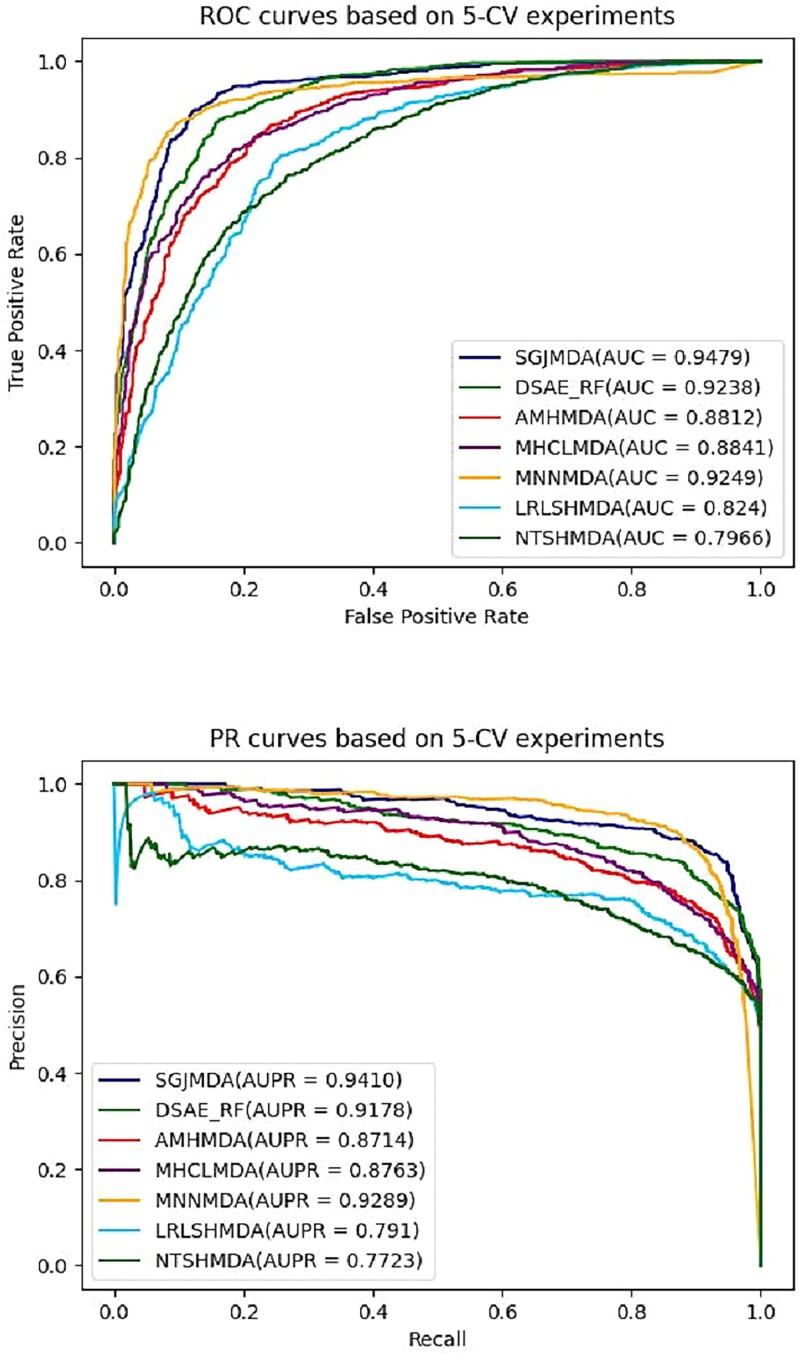
ROC and PR curves of different methods in association prediction based on 5-CV.

We plot the ROC and PR curves of these methods based on 5-CV tests in Fig. 6 for comparison. It can be found that the average AUC value of SGJMDA is 0.9479, which is 2.41% (DSAE_RF), 6.68% (AMHMDA), 6.38% (MHCLMDA), 2.30% (MNNMDA), 12.39% (LRLSHMDA), and 15.12% (NTSHMDA) higher than the other 6 methods, respectively. We can also see the average AUPR value for SGJMDA is 0.9410, which is 2.32% (DSAE_RF), 6.96% (AMHMDA), 6.45% (MHCLMDA), 0.54% (MNNMDA), 14.94% (LRLSHMDA), and 16.83% (NTSHMDA) higher than other methods, respectively. The results of other performance indicators based on 5-CV tests are shown in Table 2. These results suggest that SGJMDA outperforms the other six methods, significantly.

**Table 2:** Performance comparison with the baseline methods based on 5-CV

Method	Accuracy	Precision	Recall	F1-score
SGJMDA	**0.8836**	0.8507	**0.9318**	**0.8890**
DSAE_RF	0.8484	0.8490	0.8482	0.8482
AMHMDA	0.7742	0.8379	0.6918	0.7467
MHCLMDA	0.7178	0.7788	0.8635	0.8187
MNNMDA	0.8775	**0.8861**	0.8666	0.8762
LRLSHMDA	0.7683	0.7324	0.8504	0.7860
NTSHMDA	0.7076	0.6559	0.8780	0.7504

**Figure 7:**
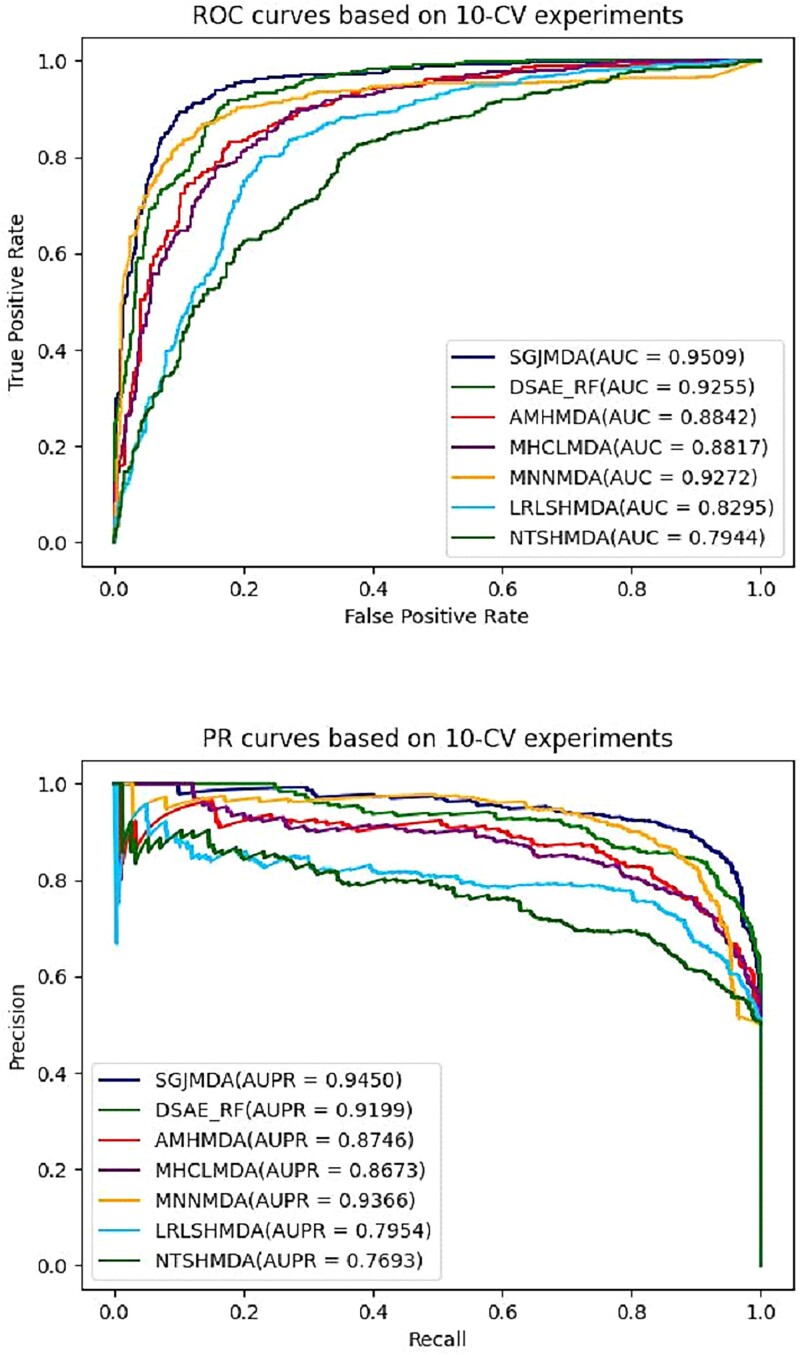
ROC and PR curves of different methods in association prediction based on 10-CV.

Similarly, we plot the 10-CV results in Fig. 7. The average AUC value of SGJMDA is 95.09%, which surpasses the other six methods by 2.55% (DSAE_RF), 6.67% (AMHMDA), 6.92% (MHCLMDA), 2.37% (MNNMDA), 12.14% (LRLSHMDA), and 15.65% (NTSHMDA), respectively. Meanwhile, the average AUPR value of SGJMDA is 0.9450, which is 2.51% (DSAE_RF), 7.04% (AMHMDA), 7.73% (MHCLMDA), 0.87% (MNNMDA), 14.89% (LRLSHMDA), and 17.51% (NTSHMDA) higher, respectively. Other performance indicators are provided in Table 3. Results from 10-CV tests again confirm the superior performance of our method SGJMDA.

**Table 3:** Performance comparison with the baseline methods based on 10-CV

Method	Accuracy	Precision	Recall	F1-score
SGJMDA	**0.8914**	0.8677	**0.9251**	**0.8951**
DSAE_RF	0.8481	0.8486	0.8480	0.8477
AMHMDA	0.7974	0.8264	0.7565	0.7870
MHCLMDA	0.7295	0.7723	0.8844	0.8237
MNNMDA	0.8803	**0.8835**	0.8789	0.8801
LRLSHMDA	0.7723	0.7304	0.8680	0.7923
NTSHMDA	0.7175	0.6706	0.8595	0.7526

**Figure 8:**
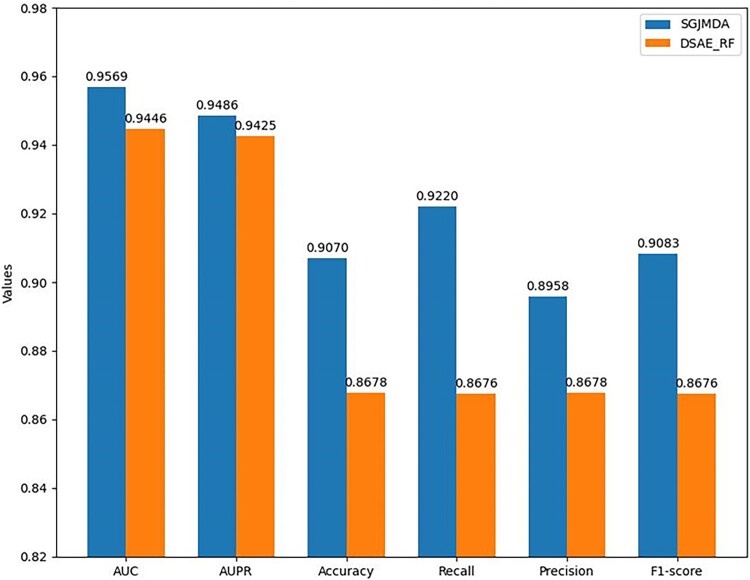
Performance comparison between SGJMDA and DSAE_RF when using the same *k*-means clustering for negative sample selection.

In addition, we adopt the same strategy of selecting negative samples as DSAE_RF [19] (*k*-means clustering selection), followed by 10-CV tests, and compare the prediction performance with DSAE_RF. The results are listed in Fig. 8. Results from Table 3 and Fig. 8 show SGJMDA receives better prediction performance than DSAE_RF [19] when using *k*-means to select negative samples.

### Case studies

We further carry out case studies on three important diseases (i.e. obesity, Crohn’s disease and colorectal cancer) to test SGJMDA’ s prediction ability in real situations. Specifically, we first exclude the association information for each specific disease from the benchmark datasets. Then, we train SGJMDA to infer disease-associated microbes. Finally, we select the top 20 predictions for validation. We use the latest versions of HMDAD, Disbiome, and Peryton to confirm the results.

As an epidemic worldwide, obesity increases the incidence of diabetes, heart disease, high blood pressure, and cancer [32]. Conventional knowledge suggests that behaviors that lead to overeating and inactivity are the main contributors to obesity. However, there are several microorganisms that have been linked to obesity in humans [33]. The findings suggest that obesity has a microbial factor, which may have potential therapeutic implications [34]. We use SGJMDA to infer obesity-associated microbes. We select the top 20 predictions and discover that 19 of them have been confirmed in the HMDAD, Disbiome and Peryton databases. We showcase the results in Table 4.

**Table 4:** The top 20 predicted obesity-associated microbes

Ranking	Microbe	Evidence
1	Corynebacterium	PMID:30654751
2	Peptostreptococcaceae	PMID:30572569
3	*Streptococcus gordonii*	PMID:19587155
4	Ruminococcus	PMID:31399369
5	Ruminococcaceae	PMID:29280312
6	Eubacterium	PMID:23055155
7	*Bacteroides eggerthii*	PMID:29388394
8	Coprococcus	PMID:30572569
9	Prevotella	PMID:31024514
10	Faecalibacterium	PMID:23985870
11	Lactobacillus	PMID:23631345
12	Streptococcus	PMID:29576948
13	Bacteriodes uniformis	PMID:29338886
14	*Collinsella aerofaciens*	NA
15	*Streptococcus oralis*	PMID:29520825
16	Proteobacteria	PMID:30386323
17	Bifidobacterium	PMID:29280312
18	Escherichia	PMID:23055155
19	Blautia	PMID:31530820
20	*Prevotella melaninogenica*	PMID:19587155

Note: NA indicates not available.

For Crohn’s disease [35], we first remove the association information from the benchmark datasets, and apply SGJMDA to predict its related microbes. We find that all the top 20 predictions are validated by the latest databases. We list the results in Table 5.

**Table 5:** The top 20 predicted Crohn’s disease-associated microbes

Ranking	Microbe	Evidence
1	Akkermansia	PMID:28222161
2	Ruminococcaceae	PMID:25121355
3	Prevotella	PMID:24013298
4	Alistipes	PMID:20816835
5	Faecalibacterium	PMID:17119388
6	Corynebacterium	PMID:22068912
7	Lactobacillus	PMID:17897884
8	*Faecalibacterium prausnitzii*	PMID:19235886
9	Ruminococcus	PMID:22068912
10	Fusobacterium	PMID:30927743
11	Bifidobacterium	PMID:26789999
12	Coprococcus	PMID:30478724
13	Blautia	PMID:31899727
14	Collinsella	PMID:20816835
15	Megasphaera	PMID:27083382
16	Rothia	PMID:26288001
17	*Collinsella aerofaciens*	PMID:26804920
18	Pseudomonas	PMID:26574491
19	Anaerostipes	PMID:26313691
20	Streptococcus	PMID:30545401

Similarly, for colorectal cancer [36], we apply SGJMDA to predict its potentially associated microbes. For the predicted 20 predictions, we find that 11 associations are confirmed (Table 6).

**Table 6:** The top 20 predicted colorectal cancer-associated microbes

Ranking	Microbe	Evidence
1	Micrococcus	PMID:28600626
2	*Erysipelotrichaceae incertae sedis*	NA
3	Aggregatibacter	PMID:27742762
4	Arthrospira	PMID:25150117
5 6	*Alloscardovia* *propionicimonas*	NA NA
7	*Peptostreptococcaceae incertae sedis*	PMID:22114001
8	Pandoraea	PMID:35672730
9	*Mycobacterium tuberculosis*	PMID:36183156
10	*Delftia tsuruhatensis*	NA
11	*Propionimicrobium lymphophilum*	NA
12	*Varibaculum cambriense*	NA
13	Acidovorax	PMID:36717544
14	Pseudothermotoga	NA
15	*Lactobacillus taiwanensis*	PMID:29650970
16	*Anaerococcus tetradius*	NA
17	Wolbachia	NA
18	Rhodobacteraceae	PMID:37317301
19	Mycoplasma	PMID:37772998
20	Treponema	PMID:35664963

Note: NA indicates not available.

Moreover, we use SGJMDA to make comprehensive microbe-disease association predictions based on the whole information in the benchmark datasets. We select the top 20 predicted results for validation. We search PubMed (https://pubmed.ncbi.nlm.nih.gov/) for confirmation, and discover that 14 predictions have been verified (Table 7).

**Table 7:** The top 20 predictions by SGJMDA

Ranking	Microbe	Disease	Evidence
1	Blautia	Chronic kidney disease	PMID:33101877
2	Micrococcus	Colorectal cancer	PMID:28600626
3	Alistipes	Chronic kidney disease	PMID:37809388
4	Klebsiella	Chronic kidney disease	PMID:37284390
5	Sutterella	Chronic kidney disease	PMID:36718700
6	Fusobacterium	Chronic kidney disease	PMID:33435396
7	Erysipelotrichaceae incertae sedis	Colorectal cancer	NA
8	Aggregatibacter	Colorectal cancer	NA
9	Odoribacter	Chronic kidney disease	PMID:37011727
10	Megasphaera	Chronic kidney disease	PMID:34357944
11	Oscillibacter	Chronic kidney disease	PMID:32560104
12	Arthrospira	Colorectal cancer	PMID:35946342
13	Staphylococcus	Chronic kidney disease	NA
14	Veillonella	Chronic kidney disease	PMID:38095826
15	Lachnospiraceae	Chronic kidney disease	PMID:37065213
16	Coriobacteriaceae	Chronic kidney disease	PMID:33681383
17	Alloscardovia	Colorectal cancer	NA
18	Propionicimonas	Colorectal cancer	NA
19	Proteobacteria	chronic kidney disease	PMID:29444477
20	Lachnospiraceae incertae sedis	Chronic kidney disease	NA

Note: NA indicates not available.

## Discussion and conclusions

Studies have demonstrated that human microbiome has a profound impact on health. Their differences in abundance and diversity can help explain the susceptibility or resistance to certain diseases. Identifying disease-associated microbes would therefore boost our understanding of the pathogenesis of diseases and promote treatment to diseases. In this study, we develop a deep learning-based computational framework SGJMDA to infer new microbe–disease associations. Comprehensive experiments, including ablation tests, comparison with other methods and case studies, are carried out. Results show the superiority of our method in association prediction.

The factors that lead to the good performance of our method can be summarized as follows. First, we use a nonlinear strategy for similarity fusion. Comparative results show the similarity fused by our method can generate more accurate predictions. Second, we apply both GCN and jumping knowledge network to exact features from microbes and diseases, which can obtain high-order neighborhood representation information for them. Finally, we calculate linear correlation coefficients not matrix multiplication as prediction scores. Ablation tests demonstrate prediction performance can be improved by calculating linear correlation coefficients as prediction results.

Although our method SGJMDA performs well in terms of prediction performance, there are still some limitations. For example, the number of experimentally verified microbe-disease associations is limited, which would affect the prediction performance. We expect to solve this issue by integrating more reliable association information discovered in the future in our model. Meanwhile, optimizing the hyperparameters in SGJMDA is also a challenging task, which is a common problem in deep learning methods. These issues are further directions we need to study.

**Key Points**

- We apply a non-linear strategy for similarity fusion for both microbes and diseases.
- SGJMDA can effectively extract embeddings for both microbes and diseases using GCN and jumping knowledge networks.
- SGJMDA outperforms existing methods and improves prediction accuracy in association prediction.

## Data Availability

The benchmark datasets and source codes used in our study are freely accessible at https://github.com/IamChenHailin/SGJMDA.

## Author Biographies

Hailin Chen, PhD, is an associate professor at School of Information and Software Engineering, East China Jiaotong University. His research interest includes data mining and bioinformatics.

Kuan Chen is a graduate student at School of Information and Software Engineering, East China Jiaotong University. His research interest is deep learning and bioinformatics.
